# A Bluetooth Low Energy Indoor Positioning System with Channel Diversity, Weighted Trilateration and Kalman Filtering

**DOI:** 10.3390/s17122927

**Published:** 2017-12-16

**Authors:** Vicente Cantón Paterna, Anna Calveras Augé, Josep Paradells Aspas, María Alejandra Pérez Bullones

**Affiliations:** 1 Department of Telematics Engineering, Universitat Politècnica de Catalunya, 08034 Barcelona, Spain; vcantonpaterna@gmail.com (V.C.P.); josep.paradells@entel.upc.edu (J.P.A.); mapb1989@gmail.com (M.A.P.B.); 2 Fundació i2CAT, 08034 Barcelona, Spain

**Keywords:** BLE, BLE Tag, trilateration, frequency diversity, Kalman filtering, indoor positioning system, accuracy

## Abstract

Indoor Positioning Systems (IPS) using Bluetooth Low Energy (BLE) technology are currently becoming real and available, which has made them grow in popularity and use. However, there are still plenty of challenges related to this technology, especially in terms of Received Signal Strength Indicator (RSSI) fluctuations due to the behaviour of the channels and the multipath effect, that lead to poor precision. In order to mitigate these effects, in this paper we propose and implement a real Indoor Positioning System based on Bluetooth Low Energy, that improves accuracy while reducing power consumption and costs. The three main proposals are: frequency diversity, Kalman filtering and a trilateration method what we have denominated “weighted trilateration”. The analysis of the results proves that all the proposals improve the precision of the system, which goes up to 1.82 m 90% of the time for a device moving in a middle-size room and 0.7 m for static devices. Furthermore, we have proved that the system is scalable and efficient in terms of cost and power consumption. The implemented approach allows using a very simple device (like a SensorTag) on the items to locate. The system enables a very low density of anchor points or references and with a precision better than existing solutions.

## 1. Introduction

By 2019, the estimated value of the indoor positioning market will increase to $4.4 billion [1]. The possibility of tracking people’s paths in commercial areas or knowing the position of a given asset in an industrial building is becoming of interest for many companies.

Regarding the challenges that positioning systems face in indoor environments, there are many factors that might impair the propagation of the signals. According to [2] these issues are mainly caused by:

- Reflection and diffraction around objects (including walls and floors) within the rooms that can cause multipath and fading effects respectively.
- Transmission loss through walls, floors and other obstacles.
- Channelling of energy, especially in corridors at high frequencies.
- Motion of persons and objects in the room, including possibly one or both ends of the radio link.

There are several approaches for Indoor Positioning Systems (IPS): WiFi-based positioning systems (WPS), Bluetooth Low Energy (BLE) solutions, Radio Frequency Identification (RFID)-based systems and Ultra-Wide Band (UWB) or Visible Light Communication (VLC) technologies [3,4]. The topic we present in this paper is related to wireless technologies.

We can use either the Time of Flight (TOF), or the Received Signal Strength Indicator (RSSI), to estimate the position of the device being tracked. The current work will focus on Received Signal Strength Indicator (RSSI). The RSSI can be measured from periodic broadcasted signals (like for example beacons from a WiFi access point or a BLE device) or from frames transmitted in unicast. If the transmitted power is known, once the RSSI is measured, it is possible to estimate the propagation losses. Then applying a propagation loss model we can infer the distance from the sender and the receiver.

In IPS there are three important factors to take into account: the arrangement of the transmitters and receivers, the RSSI analysis and the wireless technology (e.g., WiFi, BLE) that will be used for the deployment.

Regarding the arrangements there are two possible scenarios, one based on a set of reference points or anchors that transmit beacon signals to the device to be located, and another where the anchors receive the signals from the devices to track. The first arrangement is more complex since it means that the devices to be tracked have to measure the signals from the anchors and transmit back the location information. Nevertheless, this solution is the most commonly used, since in many applications the object to be tracked is a person equipped with a mobile phone.

The RSSI analysis can be done in two main ways: by mapping the radio propagation losses to distance according to the propagation model, and by means of fingerprinting techniques.

In the first case, we consider that the signal is transmitted according to the propagation model and we get the distance by finding and isolating it on the model and solving the equation [5,6,7,8,9,10,11,12,13,14,15,16,17]. The main problem with this approach is the difficulty of choosing which indoor propagation model is the most appropriate (free space path loss model, Log-Distance Path Loss model, Log-Distance Path Loss model with shadowing or two-ray ground reflection model, among others). The disadvantage of this method is that with the distance from one reference point we offer a very poor precision on the location, although it is possible to improve it by estimating the distance to several ones and applying trilateration techniques.

In the second case, the fingerprinting method [18,19,20,21,22,23,24,25,26,27,28,29,30,31,32], several measurements are taken at each possible position, so that there is a pre-established map of distance-RSSI values. With this approach the accuracy of the system is greater compared to the previous one, but we need an exhausted characterization of the environment. Furthermore, it is very susceptible to any change, which causes having to characterize the medium again.

Both, the arrangement of the transmitters and receivers, and the RSSI analysis, should be chosen to best fit the wireless technology. The most popular technologies used for IPS are WIFI, BLE, RFID and UWB, and they all have their pros and cons:

- In the case of WiFi solutions, the main advantages are: (a) they are already deployed in many places, so there is no need for a new network infrastructure, and (b) they have a long range compared to the other solutions. The main drawback of WiFi solutions lies in its poor accuracy, from 5 m to 15 m when using fingerprinting. In order to increase the accuracy more access points are needed which increases the cost of the deployments.
- In the case of BLE technology, its main strength lies in its low cost and low power consumption, even though with an acceptable accuracy (1 m error). However, this technology usually needs additional equipment (deployment of a BLE network) and it has a short range, up to 20–30 m.
- In the case of RFID systems, its accuracy is the best among all the technologies (error below 0.1 m) within its lifetime (no battery needed). Its main drawback is the short range (below 1 m) and the extensive and expensive installation of large amount of readers to cover large areas.
- In the case of UWB technology, the most important features are: (1) its accuracy (error below 0.3 m); and (2), its range, up to 150 m, which is the highest among the technologies presented here. However, its main disadvantages are: (1) high power consumption; and (2) high cost.

Although WiFi solutions have been popular in the past, BLE devices offer such a low cost and low power alternative [5], that they have become attractive for places that do not offer WiFi infrastructure. WiFi does make possible the transmission of high data rates, however, the type of data typically required to be sent for a positioning system does not require high throughput, therefore BLEs offering of 1 Mbit/s for their data transmission are acceptable. Consequently, BLE represents a cheaper and more energy-efficient option to implement indoor-outdoor detection and position applications (by means of systems like BlueDetect [33]) than WiFi solutions.

Fast fading or multipath effects have a special impact on the propagation of BLE signals, since the indoor environments contribute to RSSI fluctuations. In [18], an analysis that shows that the BLE signals suffer from a −30 dBm drop caused by the multipath effect was presented. Focusing on this fact, the multipath attenuation is not constant and it can vary at any distance without any pattern. All of the effects mentioned before make it difficult to model an indoor mobile radio channel, because the channel varies significantly with the environment. Therefore, in order to use the relationship between distance and RSSI for and IPS, it will be necessary to understand and deal with all the fluctuations of the signals so that a precise system can be implemented.

As regards IPS with BLE, we should take into account that this technology usually uses RSSI to estimate the location. The authors in [6] detailed the reliability of this assumption. They compared a set of different mathematical methods, such as moving average method or weighted average method, with a theoretical reference curve, in order to verify the reliability of RSSI measurements. Their experiments show that considering RSSI values as the only input to reliably compute the location of a node is not enough. In addition, they state that when using trilateration techniques, both antennas, sender and receptor, must be isotropic.

In this paper, we propose and present an implementation of an IPS that uses BLE technology with CC2650 SensorTags [34,35] as the devices sending BLE beacons, Raspberry Pi as receivers with Adafruit sniffers [36] to get the BLE beacons, and a server platform in charge of processing the distances. Our main goal with this implementation is to develop a complete IPS with low cost and high accuracy independently of the environment the system is placed on. In order to do so, we have defined and implemented the following features:

- The use of *channel diversity* as a way of mitigating the effect of fast fading, as well as the effect of interferences during RSSI measurements. Instead of choosing only one BLE communication channel, we use the three BLE advertisement channels (Channel 37, Channel 38 and Channel 39) available to send BLE beacons. They are sent in small lapses of time (the three advertisements in 3 ms intervals), so that channel characteristics are quite the same, and then the effect of fast fading can be minimized by combining them. After that, we compute the channel having the best accuracy in terms of distance-RSSI, and use that one for positioning calculations. By the knowledge of the authors there is no other proposal using this approach and achieving such an accuracy.
- The use of a *trilateration method based on weights*. Trilateration works perfectly when the measurements taken converge to a single point. However, in most of the cases we have an area of possible locations instead of a single location point. Our proposal improves the accuracy of trilateration by considering as more reliable the information provided by the closest receivers to the sender and move the estimated position to the position that receiver suggests.
- The use of *Kalman filtering (KF)* to avoid incoherent computation of the location. Sometimes, we may get wrong RSSI measurements leading to wrong and very unlikely estimated positions. KF is a well-known method to help reduce the impact of wrong measurements on the system.

The estimation of the cost in an IPS is affected by several factors. There is the infrastructure, the installation and the maintenance costs. In the most commonly used applications, where the item to be tracked is a person, or better said the smartphone of a person, the infrastructure to be deployed is a set of beacons or anchors that can be battery powered and that do not require any connectivity. In this use case, the device tracked is costly, requires connectivity and the batteries have to be recharged quite frequently. If this solution is adopted to track objects, we can say that the infrastructure cost depends on the area to provide service and the number of objects or items to be tracked. A density of one beacon every 20 square meters with a precision between one and two meters is common. The installation cost is low since no connection or main power is required, but the maintenance cost is high since the recharging of batteries in the objects to be tracked is required. Moreover, the calibration of the propagation environment is needed (it is common to use fingerprinting). In the system we propose, the cost of the infrastructure depends on the coverage area and the number of devices also, but in a different manner. In our case, the device to be tracked is similar in cost to the beacons used in the previous example, and ranges from 15€ to 30€ depending on the battery size (the system has low power consumption). The devices that are fixed and receive the beacons require three BLE radio interfaces and a system with Ethernet or WiFi connectivity. For our setup, the cost is around 150€ per device, assuming that they are connected to the power mains. The density of these devices that are fixed and receive the beacons can be low, due to the radio processing technique we employ (BLE), being around one node every 200 square meters for a precision of one to two meters. The system does not require frequently recharging the battery, and no periodic calibration is needed. Using a different radio technology for implementing tracking is possible. One interesting candidate is UWB, specifically the one based on IEEE802.15.4a. In this case, and assuming the most common method, the device to be tracked requires a device that costs between 100€ and 150€, and a set of fixed devices that should be powered and connected to the network, with an estimated cost of 120€ each. The density of fixed devices is of one every 600 square meters. The precision in this case can be between 0.5 and one meters. The system does not require calibration, but, as the power consumption is significantly higher than with BLE, the battery of the devices to be tracked must be replaced/ recharged quite frequently. The figures we have provided show that if we have a large number of devices to be tracked, which should work unattended our proposed solution is the best in terms of cost.

The rest of the paper is organised as follows: Section 2 presents the state of the art of WiFi and BLE IPSs. In Section 3 we detail our implementation. In Section 4 we discuss the used methodology and the theoretical basis of the implementation. Also in Section 4 we present the tests carried out in the different scenarios, and the corresponding results. Finally, we present the conclusions of the study, summarising the performance of the system, in Section 5.

## 2. Related Work

Most of the literature refers to systems using either BLE or WiFi technologies, since they are the ones that perform better in terms of quality and cost. Furthermore, the most common way to estimate the position of a device in almost all the systems is by means of RSSI. In the following sections, we present the related work taking into account the main technique used in each paper and the comparison with our proposal. In most of the research works, beacons refer to the fixed anchors or references that send the beacon signals to be received by the device to be tracked. In our proposal, beacons are the devices that we want to track, and that send the beacon signal.

### 2.1. Fingerprinting Approaches

One of the most common methods of positioning is by using a fingerprinting algorithm. A first approach of IPS with BLE and this technique is described in [19]. In the paper, the authors describe a massive deployment of fixed BLE beacon devices in a room, with around 0.8 m distance between them, to ensure a good location of the mobile node being monitored. The main goal of the research was to be able to decide if a BLE device is in the room or not. Although they achieve this objective, we consider a disadvantage that the cost of the deployment is high, and that an RSSI template matching method like fingerprinting is used. The average result between device position and estimated position is 2.4 m.

In [20], the authors proposed another system using the fingerprinting technique. Their system achieves a position error lower than 1.58 m when walking from a fixed position to another. It uses a smartphone as the device to capture the BLE beacons. The main drawback of this solution is the fingerprinting method, which requires intensive offline measurement to characterize the environment. There is another approach where authors use fingerprinting [21], reaching an estimated error of 4.12 m 90% of the time. Those results are not good if we take into account the extensive characterization of the environment that is needed. Another conclusion they reach to is that the orientation of the BLE device (i.e., the directivity) is an important factor.

In [29], the RSSI of the different channels is processed separately, and its data is kept in a database that was later used for fingerprinting. The scenario used was a corridor.

The authors in [22] propose a combination scheme of BLE and WiFi fingerprinting obtaining an enhanced estimation position. Results show an accuracy of 2.33 m at the cost of having to deploy both BLE and WiFi networks. In [23] we find the opposite: WiFi fingerprinting makes the roughly position estimation and the BLE is in charge of enhancing the accuracy of the system. The results are promising at the cost of duplicate BLE and WiFi devices.

In [37] the authors make a comparison between BLE with particle filtering and fingerprinting. Their results show that fingerprinting outperforms particle filtering in a 1.5 m × 12 m corridor, but particle filtering outperforms fingerprinting with an accuracy of less than 4 m in 90% of time in an 8 m × 6 m room.

In [25], the authors monitored the forty BLE channels using a spectrum analyser. A BLE scanner was also used to listen to the three advertisement channels. They used fingerprinting-based techniques and the tests were performed in two environments: an anechoic chamber and an office. The conclusion they achieved was that the results for both scenarios were very similar.

There are other authors who analyse the number of devices that should be deployed to improve the accuracy of the IPS system. In [26], the authors provided a study of BLE fingerprinting using beacons distributed around 600 m^2^ to calculate the position of a device. In this case, the conclusion is that the more beacons were deployed, the more accurate the calculated positions are.

In [30] the advertisement channels are treated separately when the measurement of the RSSI is done. The authors develop a separate channel advertising scheme to measure RSSI on each advertisement channel. The technique consists in putting a mask on two of the three channels. For this research, the fingerprinting method was also used, an individual database for each of the advertisement channels was build. In the estimation stage, the RSSI values captured (one for each channel) were compared to the ones kept on each of the individual databases, in order to estimate the locations. The RSSI used to build the database were selected by calculating the mean of a series of RSSI values captured in the desired position. Tests were performed in corridors where four beacons and two receivers were deployed. The results showed a high number of errors due to the fact that, with the fingerprinting technique, the position can only be calculated if the user is inside the positions saved on the database. The authors indicated that the system proposed had an accuracy of 2.56 m at 90% of the time with one beacon each 9 m. The negative side of this study, and all the ones that use the fingerprinting method, is the fact that a characterization process needs to be performed to build the database, as also shown in [31], where authors include deep learning with its corresponding training phase.

In [28] the authors conclude that the number of BLE beacons is crucial for accuracy purposes. Moreover, they compare the accuracy of using only one BLE advertising channel and the accuracy when using the mean over all the channels, to conclude that considering the information of all the channels increases the probability of a good position estimation. In [32], researchers try to solve the problem of environment characterization for fingerprinting approaches. To do so, they use what they call Dynamic RSS feedback algorithm to characterise the environment, using part of the measurements. They achieve an estimation error of 1.5 m in a cafeteria without considering obstacles, and 2.3 m when considering them. Researchers in [24] highlight the importance of the transmission power settings on beacons, and how changing them can help avoiding refractions and multipath. Another paper concluded that there is an important relationship between the number of installed beacons and their positioning with the accuracy of the system [27]. The authors estimated a practical path loss model using four beacons. The measurements were taken during a minute at different reference distances from one to 13 m. For the distance calculation, the Log-Distance Path Loss model not considering the shadowing effects was used. The path loss exponent was characterised for the environment.

In [18], the authors carried out a more theoretical analysis also using a fingerprinting method. They provided a comparison between the BLE and WiFi fingerprinting methods considering physical aspects. Tests were performed in a corridor, where no obstacles were present. To mitigate the multipath effects, the mean of the RSSI values of the captured data were calculated. They concluded that processing data from different channels gave better results than doing it with only one. They demonstrated that BLE fingerprinting method achieves an accuracy of less than 2.6 m in 95% of the time when walking in a room, much better compared to WiFi accuracy, which is about 8.5 m, 95% of the time. Again, the authors reveal the most important drawback of any fingerprinting method: degradation of the performance over time due to environment changes.

### 2.2. Non-Fingerprinting Approaches

In [7], the authors analyse the energy consumption of using BLE tags as beacons, and conclude that it is one of the main advantages of the devices. They also consider the necessity of post-processing the RSSI measurements due to the dropping accuracy for distances larger than 3 m. Another interesting study is done in [38], where the authors compared the accuracy of signals at different frequencies (WiFi 2.4 GHz, BLE channel frequencies and 5 GHz). The goal is to detect the floor where a user is, and to estimate his/her position, but the results are poor, especially for 5 GHz signals. The researchers in [8] describe the problem related to RSSI sensibility to receivers, and propose a location scheme based on advertising beacons with different transmission power levels to improve the coverage of the signal and to decrease the location error. 

In [9], the authors propose an equation to enhance the accuracy of the distance when BLE beacons are used. Instead of using the already known propagation models, the authors take the RSSI values captured and, accordingly, they implement a fitted distance curve to perform the distance calculation. The equation they propose is only useful for the analysed environment. The results were not favourable since they were not able to obtain accurate positions.

An example of a commercial indoor positioning system is presented in [10], in which the authors point out it to be an accurate and a robust positioning and tracking system using commercial mobile devices with an integrated feature of route finding. In the analysis, the beacons with the strongest RSSI measurements from a grid are taken. The data is saved on the database (MAC and position of the beacon). Iterative Least Squares Trilateration (LST) is applied to obtain the position. The disadvantage of this proposal is that it requires a learning phase.

A different technique called stigmergy was introduced in [11]. The principle is that the trace left in the environment by an action stimulates the performance of the next action. Applied to IPSs, it takes into account the previous states of different nodes being tracked to estimate the current position of the present node. It also uses the Min-Max localization algorithm. This algorithm maximizes the minimum gain of a system when we do not know how the system will behave. In this case, it minimises the positioning error when we have fading in the channel. Min-Max combined with the mentioned stigmergy method leads to acceptable location accuracy with a position error lower than 1.8 m during 75% of the time when moving around a room. However, the solution uses of a large amount of BLE beacon devices, increasing the overall cost of the solution.

In [12], the key point is the use of several antennas for RSSI stabilization. Taking into account the high dispersion on RSSI values due to refractions, fading and shadowing, among others, the research considers the use of multiple antennas to mitigate this problem. Furthermore, the research states some interesting RSSI combination techniques such as Equal Gain Combiner, i.e., the mean of the RSSI obtained at the different antennas, or the Maximum Ratio Combiner (MRC), which weights each RSSI depending on its signal quality. The research concludes that this method, MRC, is the one achieving the best results on RSSI stability.

In [13], its authors introduced a KF to improve the accuracy in the position calculation. The authors develop an Android app to locate a user inside a building. The key point of the research is the use of KF as a way to reduce RSSI fluctuations. The authors state that KF greatly helps to obtain a stabilized RSSI they use to accurately locate the user. However, they are still unable to locate the exact position of the user inside a room.

Finally, we find some different approaches for indoor localization. In [17] the main proposal is adaptive ranging, a device to device communication scheme to obtain information about the environment and use the information to select the best parameters for the propagation models. Additionally, the authors introduce multi-lateration along with particle filter, to estimate the final position. The authors in [39] use BLE for activity recognition, relating the location of the user with a particular activity.

### 2.3. Similar Studies to Our Proposal

Projects that use a similar architecture to the one proposed in our paper can be found in [5,14,15]. In [5] the authors analyse the operation of BLE tags for IPSs. They conclude that their low cost makes them attractive for the deployment of IPS. The authors in [14] show the usage of CC2650 SensorTags to implement an IPS in a 12 m corridor. Their positioning calculation results offered low accuracy, but they stated that this BLE tag are ideal for IPS, since they not only allow broadcasting RSSI values, but they can also be used to control environmental variables (temperature and pressure) with low cost. In [15], the authors propose a smoothing algorithm to decrease the effects of the environment on the RSSI values by locating a device using also CC2650 SensorTags. A database with the captured RSSI data was build and used to characterise the path loss exponent to the environment. They calculated the distance to the different tags and used trilateration as the location algorithm. The testing scenario was a wide space with metal and wood obstacles in which four BLE tags were fixed. The authors stated that the accuracy of their system was high (error of less than 1.162 m). These results were obtained after doing the characterization of the environment, which is a process that complicates the deployment of the system.

The research in [16] has a similar structure than the one presented in our paper. However, the main difference with our work is that they use devices with BLE modules as advertisers and a mobile phone as a receiver. We use BLE tags as advertisers and Raspberry Pi with the same BLE modules as receivers. Another difference is that the authors propose a system using BLE modules at fixed positions within KF to filter the RSSI. In addition to filtering, they use trilateration and dead reckoning as algorithms to locate the node. Additionally, they integrate those algorithms into what they call “Kalman-based fusion”. The results of their experiments show that trilateration and Kalman-based fusion have the best performance of the three methods (trilateration, dead reckoning and Kalman-based fusion), with an error below 0.75 m. However, they consider a corridor whose first 6 m have a width of 2.3 m and a height of 2.65 m and after that the width is increased to 3.6 m, which is pretty small considering real environments, and they place the BLE modules very close to each other, increasing the final cost of the solution. Beyond accuracy, researchers also state advantages and disadvantages between the methods. Regarding precision, trilateration and fusion methods achieve a nearly constant error while when using dead reckoning, which requires no additional hardware, the error grows with time. 

### 2.4. Comparison of Related Work Studies

Table 1 summarises some of the studies presented in this section, including a comparison of some relevant research with the implementation we present in this paper.

From the research presented in this section we can reach some important conclusions. Some of the studies mentioned before characterized the environment in order to obtain a higher accuracy in their results. This characterization was done through the implementation of a fingerprinting method or through the characterization of the propagation model variables. The problem with characterization is that resulting systems can only be used in the scenario where the characterization was performed. They cannot be immediately deployed in other scenarios because a learning phase is always needed to collect all the relevant data. This means that an extra investment of time and resources will be necessary. Another important aspect that stands out in most of the researches, is that their tests were performed in corridors without obstacles where the results will always be better in absence of obstacles and with better signal propagation.

The system we propose does not require the characterization of the environment, and it has been tested in different scenarios to show that we have consistent results that can be extrapolated. Regarding the use of BLE tags over beacons in [5,14,15] we were able to see the advantages that come with the use of these devices: high accuracy, low power consumption, low price and different applications. As it was mentioned, with a BLE tag an IoT deployment that calculates the position and other environmental variables could easily be implemented.

In this paper we present an implementations of a BLE IPS that offers high accuracy in the positioning calculation, while using a low cost and energy efficient device.

## 3. Proposed IPS BLE Based System

The objective of the system proposed is to track the position of a device by means of BLE in indoor environments. In order to do so, the system places L receivers (BLE modules) in fixed positions, while the senders (BLE SensorTag modules) move around the scenario. These senders represent the devices that are being tracked. The BLE SensorTag modules act as BLE beacons.

Regarding the receivers, three measurements are needed, in order to apply the trilateration method so that a minimum of three receivers is mandatory for each room.

As we mentioned in the introduction, our main goal is to deploy a full working system with low cost and low error. We define the error e as the euclidean distance from the estimated position $p_{e}$ to the real position, $p_{r}$:

(1) $$e=\sqrt{{\left(p_{\mathrm{rx}}-p_{\mathrm{ex}}\right)}^{2}+{\left(p_{\mathrm{ry}}-p_{\mathrm{ey}}\right)}^{2}},$$

where the subindices x and y correspond to the x and y axis, respectively. To improve the accuracy, we propose the use of frequency channel diversity, a weighted trilateration method and Kalman filtering.

### 3.1. General Overview of the System

**Figure 1:**
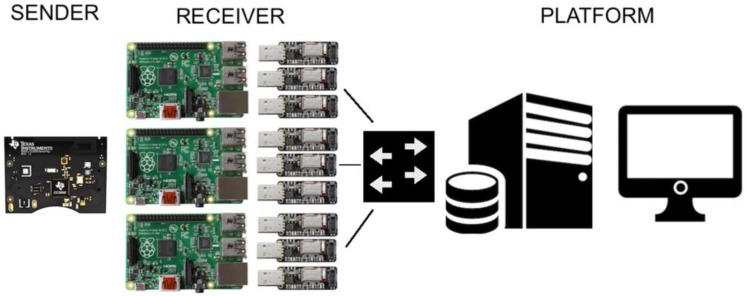
Overview of the system.

The BLE indoor positioning system is composed of the elements shown in Figure 1. The first element is one or several senders (BLE SensorTags), who send the BLE advertisements, therefore the senders are acting as BLE beacons. The second element is a set of at least three receivers. Each receiver is a Raspberry Pi with three sniffers, in order to listen the three BLE advertisements channels (Channel 37, 38 and 39). Each sniffer is programmed so it only processes one channel advertisement. Finally, a platform (server) is needed to receive the distances computed by each receiver to estimate the current position of the sender nodes (BLE beacons).

As a sender, the SensorTag CC2650 [34,35] an IoT device developed by Texas Instruments is used. It has sensors for temperature, humidity, pressure, magnetometer, gyroscope and accelerometer, which make it a powerful tool for many applications. In our system, the SensorTag CC2650 is the one in charge of sending the BLE beacons. For each time interval (time between advertisements, that can be configured), this device transmits the beacon messages (non-connectable advertisements) through each of the channels (37, 38 and 39) sequentially, within an interval of 3 ms [40]. Therefore, the changes of RSSI in each channel are only due to the frequency used. The messages sent in the same beacon interval (one for each channel) have the same counter ID so that the receiver can identify them to apply channel diversity accordingly. Every sender has a unique MAC address used to differentiate messages from different senders.

Regarding the sender, firstly we have to take into account the directivity of the antenna of the BLE Tag. Even though the datasheet shows it is almost omnidirectional, tests that we performed showed that there is a difference of up to 8 dB in some cases. For a system that fully relies on omnidirectional directivity, this is a huge drawback. Secondly, the transmission power of the device, can be set up to 5 dBm maximum, while the BLE standard allows it up to 10 dBm. When dealing with environments full of people, walls and interferences, 5 dBm are not enough, in which case we must increase the number of receivers.

The SensorTag CC2650 has been programmed using the TI Code Composer Studio to send advertisement beacons each 100 ms at maximum power of 5 dBm. Moreover, it has been programmed to detect if the device is moving or not. In the first case it keeps sending beacons at the same rate, but in the second case it changes the frequency to beacon every 5 s instead of 100 ms to save battery.

As receivers, we used Raspberry PI (RPi) with three Adafruit sniffers [36] in each, responsible for sniffing the beacons sent by the senders in channels 37, 38 and 39. As we devote one sniffer per channel, the sniffer is continuously listening the channel to decode any transmission. As every advertisement has the sequence number introduced by the sender, the time of arrival to the sniffer is not relevant to differentiate advertisements. The RPi’s are programmed in Python, to turn on the sniffers and configure them to listen to a particular channel in order to take advantage of the channel diversity. When beacons are received, the RPi runs a script, computes the distance to the sender and sends the information to the platform. In the rest of this section we detail how we obtain an accurate position from the received RSSI form the BLE beacon.

The Adafruit sniffers are low cost BLE devices. These modules are based on the Nordic nRF5182 BLE chipset with relatively poor RSSI measurement accuracy (max. ±6 dB [36]). Comparing this value with other devices, we can see that it is a common value, for example, the CC2650 BLE chipset [35] has a typical value of ±4 dB and others like the Cypress PSoC 4XX7_BLE family [41] have a typical value of ±5 dB. The poor RSSI measurement accuracy can affect the overall RSSI accuracy of the system, and is another reason to design and implement other methods than can improve the accuracy on the positioning system. Moreover, accuracy on BLE beacon power is another issue to take into account in overall accuracy. In our system both effects are not a critical issue, since both senders and receivers can be calibrated. In others systems, for example, when receivers are mobile phone based, this calibration cannot be performed.

The system uses the platform to estimate the position of each of the senders according to the information received from the receivers. First of all it creates a socket connection with each receiver, identified by its MAC address. Then it listens to the incoming information from the receiver, which sends to the platform its distance to each sender. In order to keep tracking the different senders without crossing of information, all of the senders are in turn identified by their MAC address as stated before. Thanks to this, the system is able to, both, keep the track of different devices, and use different receivers, at the same time. We use C as the programming language in the platform to maximize performance and scalability. The number of RSSI samples that we consider in each position calculation, as well as how often we update the sender position is a system parameter that can be tuned depending on the application requirements.

### 3.2. Channel Diversity

As BLE transmits in three advertisements channels (37, 38 and 39), the receiver checks the RSSI in the different channels and applies a combination scheme explained later to obtain the best results, taking advantage of channel diversity to improve the performance of the system.

In our system, the BLE Tags send advertisements for the three BLE channels, 37, 38 and 39. All these advertisements are identified by the MAC of the sender and the counter ID for channel diversity. In reception, the Adafruit sniffers capture the different frames and each RPi extracts sniffers frames, and insert them into a circular matrix for each identified sender (we use the MAC of the sender to this purpose). Table 2 shows the circular matrix of one receiver for one specific sender. The RSSI values in each row correspond to the counter ID, so that we can apply channel diversity. In each performed test we will specify the size of the matrix and how we use this matrix to estimate the position. Due to transmission errors, some rows are not completed and can have only one or two values.

#### 3.2.1. Combination Algorithms

Unlike all the approaches we reviewed in Section 2, in the present paper we will use channel diversity as a way of improving performance. We could select, either randomly or using some scheme, a particular advertisement channel (Channel 37, 38 or 39) and use it as the reference. Nevertheless, we think we can use all the information we have about the channels and combine them in either of the following ways:

- Select the one providing the biggest RSSI value (*biggest algorithm*). In this case we consider as the best channel the one whose related RSSI is the biggest among all the channels. In other words, it takes the RSSI of the channel that performs better:
  (2) $$RSSI_{max}=\max\left(RSSI_{ch\;37},RSSI_{ch\;38},RSSI_{ch\;39}\right)$$
- Take the mean between all the channels (*mean algorithm*). Now we compute the mean value of the RSSI values of the three channels. Since we are using propagation models, we could get closer to a model by taking the mean instead of only using one channel:
  (3) $$RSSI_{average}=\frac{1}{3}\overset{39}{\underset{i=37}{\sum }}RSSI_{ch\;i}$$
- Obtain a RSSI value from the Maximum Ratio Combining (MRC) algorithm (*MRC algorithm*). This approach is to weight the channel in such a way that, when combining them, we trust more the ones with bigger RSSI values than the ones with smaller values, but we still take these into account in the final RSSI computation. The RSSImin value in the numerator has been chosen according to the sensibility of the channel sniffers [36]:
  (4) $$RSSI_{MRC}=\overset{39}{\underset{j=37}{\sum }}\frac{RSSI_{j}-RSSI_{min}}{\sum_{i=37}^{39}RSSI_{i}}RSSI_{j}$$

### 3.3. Distance Estimation from RSSI

Once we have selected the optimum RSSI measurement using channel diversity, we need to calculate the corresponding distance from the sender to the receiver. We have selected three propagation models [18,42,43], looking for the most accurate one to estimate the distance (d) from the RSSI value. The first one is Log-Distance Path Loss model with shadowing showed in Equation (5), valid for distances d > d_0_:

(5) $$\mathrm{RSSI}=\mathrm{RSSI}\left(\mathrm{do}\right)+10\mathrm{nlog}\left(\frac{d}{d_{0}}\right)+X\sigma ,$$

where RSSI(do) is the RSSI at a reference distance d_0_ = 0.8 m (calculated with the free space propagation model), the parameter n is the path loss exponent and Xσ zero-mean Gaussian distributed random variable with standard deviation that attempts to compensate the random shadowing effects. The parameters n and Xσ have been chosen according to the different scenarios and following the recommendations of [42].

The second one is the International Telecommunication Union (ITU) model for indoor environments showed in Equation (6):

(6) $$\mathrm{RSSI}=20\log f+\mathrm{Nlog}\left(d\right)+P_{f}\left(n\right)-28,$$

where f is the frequency in MHz, N is the distance power loss coefficient and $P_{f}\left(n\right)$ the floor loss penetration factor. N and $P_{f}\left(n\right)$ have been chosen according to the different scenarios and following the recommendations of the ITU [43].

The third one is the empirical model shown in Equation (7):

(7) RSSI=10nlog(d)+A,

where A is the empirical measured RSSI at a distance of 1 m between sender and receiver considering Line-of-Sight (LOS), n is the path loss exponent. The empirical model relies on the characterisation of the scenario. Even though the idea of our research is not to use any type of characterisation to avoid the disadvantages it implies (constants calculation, changes on the environment, etc.), we have considered this model since it has not a strong characterisation and will provide us a reference performance level.

In our case, we compute A as the RSSI at a distance of 1 m from the sender to the receiver, which we consider to almost be invariant, as both, sender and receiver, are really close one another, and the conditions cannot affect much at that distance. A comparison between all the models is presented in Section 4.

### 3.4. Weighted Trilateration

Once we have the estimated distance from the sender and each receiver, we must next estimate the position of the BLE device. As we stated before, mathematical trilateration is not always possible since there is not always a single intersection of the three estimations but rather an area of possible locations. In this paper we present a method that calculates the location by trusting more in the measurements from the devices that are estimated to be closer to the sender. We call the method weighted trilateration. It can be used with L receivers, being L greater or equal to 3, since we need at least three distances for trilateration. When more receivers are placed, we consider only the three receivers whose calculated distance to the sender is the smallest, according to our approach of trusting more the devices closer to the sender. The use of more than three receivers improves the accuracy of the system.

Given three different measurements (i.e., distances), $r_{1}$, $r_{2}$ and $r_{3}$, from receivers R1, R2 and R3, to the sender S, we calculate the associated pair-wise weights between R1, R2 and R3 as follows:

(8) $$\{\begin{array}{c} w_{a\;b}=\frac{r_{a}}{r_{b}}\;if\;r_{a}\;<\;r_{b}\;\\ w_{b\;a}=\frac{r_{b}}{r_{a}}\;if\;r_{b}\;<\;r_{a}\; \end{array}\;with\;a=1,2,3;\;b=1,2,3\;$$

Depending on the case of trilateration we move from the initial estimated point, which is in many cases the middle point in the probable location area, to the receiver that is closer to the sender, that is, the one which provided the smaller distance d to the sender S, a distance proportional to the weights calculated in Equation (8).

In order to clarify the method, we explain the main cases we may face when dealing with location computation using trilateration with three obtained distances.

**Figure 2:**
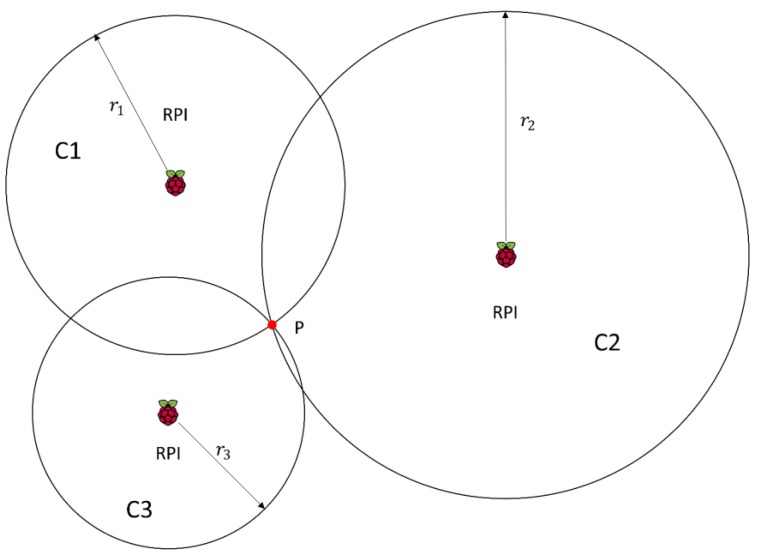
Trilateration: three circle intersection.

- Circles intersect at one single point: The ideal case is shown in Figure 2, where all the circles intersect in one point.

We can see in Figure 2 that circles *C*1, *C*2 and *C*3 intersect at point *P*. For these circles, we have the following set of equations:

(9) $$r_{1}{}^{2}={\left(x-x_{1}\right)}^{2}+{\left(y-y_{1}\right)}^{2}\;r_{2}{}^{2}={\left(x-x_{2}\right)}^{2}+{\left(y-y_{2}\right)}^{2}\;r_{3}{}^{2}={\left(x-x_{3}\right)}^{2}+{\left(y-y_{3}\right)}^{2}$$

where $r_{1}$, $r_{2}$ and $r_{3}$ are the radius of the circles *C*1, *C*2 and *C*3 respectively, and the tuples ($x_{1,}y_{1}$), ($x_{2,}y_{2}$), and ($x_{3,}y_{3}$), are the centre of the same circles. In the case we have L receivers, the set of 3 equations of (9) becomes a set of L equations from which we take the three equations related to the receivers closer to the sender.

When the three circles intersect in one point, the set of equations in (9) can be solved, leading to one single point *P* = (*x*, *y*) given by:

(10) $$x=\frac{\left|\begin{array}{cc} \left(r_{1}{}^{2}-r_{2}{}^{2}\right)-\left(x_{1}{}^{2}-x_{2}{}^{2}\right)-\left(y_{1}{}^{2}-y_{2}{}^{2}\right) & 2\left(y_{2}-y_{1}\right)\\ \left(r_{1}{}^{2}-r_{3}{}^{2}\right)-\left(x_{1}{}^{2}-x_{3}{}^{2}\right)-\left(y_{1}{}^{2}-y_{3}{}^{2}\right) & 2\left(y_{3}-y_{1}\right) \end{array}\right|}{\left|\begin{array}{cc} 2\left(x_{2}-x_{1}\right) & 2\left(y_{2}-y_{1}\right)\\ 2\left(x_{3}-x_{1}\right) & 2\left(y_{3}-y_{1}\right) \end{array}\right|}$$

(11) $$y=\frac{\left|\begin{array}{cc} 2\left(y_{2}-y_{1}\right) & \left(r_{1}{}^{2}-r_{2}{}^{2}\right)-\left(x_{1}{}^{2}-x_{2}{}^{2}\right)-\left(y_{1}{}^{2}-y_{2}{}^{2}\right)\\ 2\left(y_{3}-y_{1}\right) & \left(r_{1}{}^{2}-r_{3}{}^{2}\right)-\left(x_{1}{}^{2}-x_{3}{}^{2}\right)-\left(y_{1}{}^{2}-y_{3}{}^{2}\right) \end{array}\right|}{\left|\begin{array}{cc} 2\left(x_{2}-x_{1}\right) & 2\left(y_{2}-y_{1}\right)\\ 2\left(x_{3}-x_{1}\right) & 2\left(y_{3}-y_{1}\right) \end{array}\right|}$$

In this case there is no need to compute any weights since there is only one possible location.

**Figure 3:**
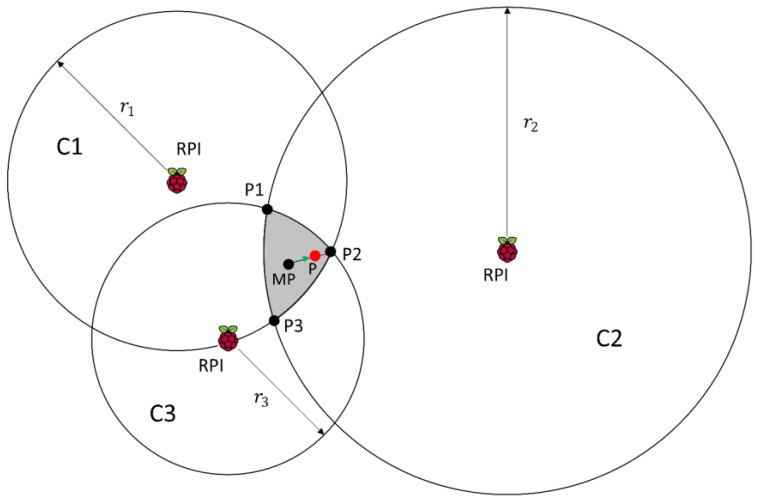
Weighted trilateration: circles intersect in an area.

- Circles intersect in an area: In the case shown in Figure 3, there is not a single point P but an area.

In order to minimize the error we propose the location as the centroid of the triangle formed by the three points *P*1, *P*2 and *P*3:

(12) $$P_{x}=\frac{P1_{x}+P2_{x}+P3_{x}}{3}$$

(13) $$P_{y}=\frac{P1_{y}+P2_{y}+P3_{y}}{3}$$

plus an adjustment based on weights. After computing the middle point between them (point MP), we take the intersection point related to the closest receivers to the device being tracked, that is, the intersection of circles *C*2 and *C*3 at point *P*2 (these circles have the minimum radius). Then, we go from MP to *P*2, the distance between them multiplied by the calculated weight using the distances of the closest receiver (RPI in circle *C*3) and the further one (RPI in circle *C*2). We consider the distance $r_{3}$ of *C*3 instead of the distance $r_{1}$ of *C*1 because, although both intersect in *P*2, *C*3 has smaller radio and therefore we trust it more:

(14) $$w_{3\;2}=\frac{r_{3}}{r_{2}}$$

(15) $$P_{x}=P_{x}+\left(1-w_{3\;2}\right)\times d\times \cos\left(\theta \right)\;P_{y}=P_{y}+\left(1-w_{3\;2}\right)\times d\times \sin\left(\theta \right)$$

being *θ* the angle of the line between points and the x axis, and *d* the distance from MP to *P*2.

Thus, when the radius of the further receiver goes to infinite, but it still intersects, the weight is 0 and we choose *P*2 as the most probable point. When we estimate the same distance from all the receivers to the sender, the weight is equal to 1 and we choose the middle point as the estimated position. Using this method we take into account the information provided by all the receivers and we rely more on the closest receiver to the device.

**Figure 4:**
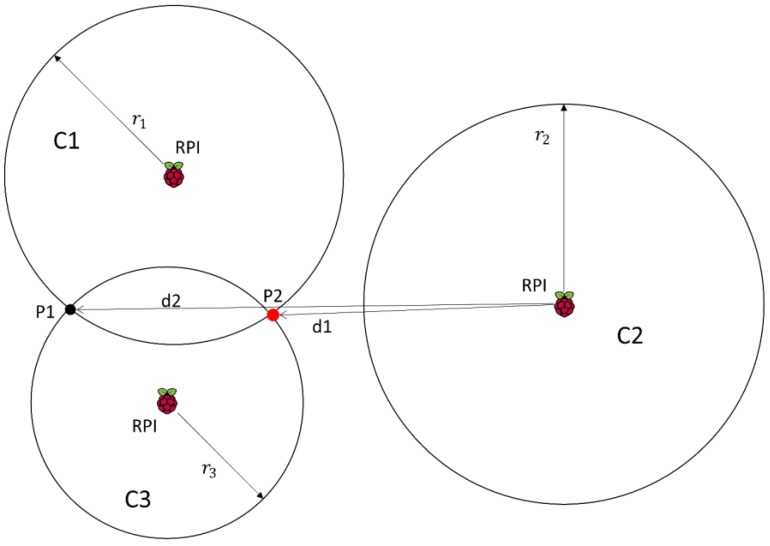
Weighted trilateration: two circles intersect in an area, one is isolated.

- Two circles intersect in an area, the other does not intersect: This case is shown in Figure 4. Analysing RSSI values, we estimate the distance from the sender and each receiver. This estimation is based on RSSI by applying a propagation loss model that provides a circular area centred at the receiver. The RSSI can be affected by multipath, and so the receivers can estimate the sender is closer that it really is, therefore, circles may not intersect.

Again we face a situation in which there is not a single point. Furthermore, now choosing the middle point between *P*1 and *P*2 as the best estimation is not the optimal solution, because we would not take into account the measurements of the receiver on circle *C*2. Instead, we calculate points *P*1 and *P*2. Then, we obtain:

(16) $$x=\frac{1}{d}\left(C3_{x}-C1_{x}\right)\pm \frac{h}{d}\left(C3_{y}-C1_{y}\right)+C1_{x}$$

(17) $$y=\frac{1}{d}\left(C3_{y}-C1_{y}\right)\pm \frac{h}{d}\left(C3_{x}-C1_{x}\right)+C1_{y}$$

where:

(18) $$d=\sqrt{{\left(C1_{x}-C3_{x}\right)}^{2}+{\left(C1_{y}-C3_{y}\right)}^{2}}$$

(19) $$l=\frac{r_{1}{}^{2}-r_{3}{}^{2}+d^{2}}{2d}$$

(20) $$h=\sqrt{r_{1}{}^{2}-l^{2}}$$

and $r_{1}$, $r_{3}$ are the radius of *C*1 and *C*3 respectively and $Ci_{x}$, $Ci_{y}$, are the coordinates *x* and *y* of the centre of the circle *Ci*.

Once *P*1 and *P*2 are calculated, the distance between the centre of *C*2 and these points is computed (*d*1 and *d*2 in Figure 4). Finally, the location chosen as the best one is the point whose distance to the radius of the circle *C*2 is smaller. In this way, not only do we consider measurements provided by *C*1 and *C*3, but we also use the information provided by *C*2. Point *P*2 is more likely to be the right one than *P*1, since *P*2 is closer to *C*2 than *P*1. In this case, we do not consider weights because there is no closer point to *C*2 than *P*2, so we do not need to compute the weighted position.

- Circles do not intersect: In this case, we get three distances whose related circles around the receiver do not intersect. As explained before, the position I is estimated based on RSSI by applying a propagation loss model that provides a circular area centred at the receiver. The RSSI can be affected by multipath, and so the receivers can estimate the sender is closer that it really is, therefore, circles may not intersect.

**Figure 5:**
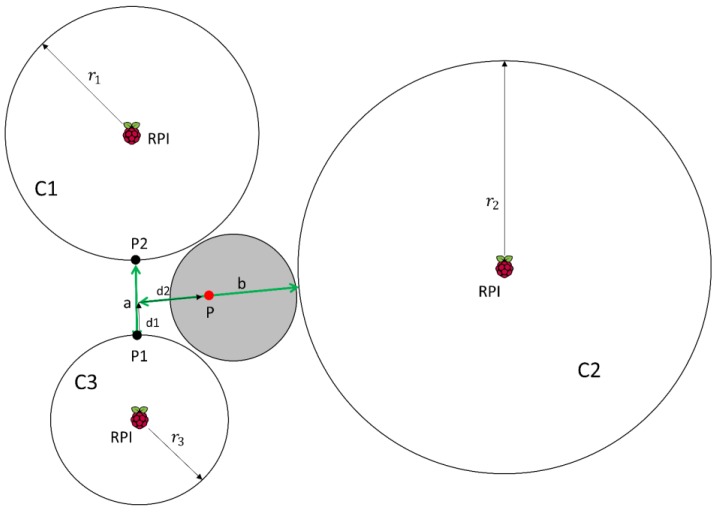
Weighted trilateration: circles do not intersect.

As it can be seen in Figure 5, the sender will be placed somewhere in the middle. In order to compute the location, we may start from the closest receiver and move towards the other receivers depending on the radius of them and the distance between receivers.

To compute the location of the sender (point *P*), we first compute the weights of *C*2 and *C*1 respect to *C*3, since it is the more reliable one (smaller radius):

(21) $$w_{3\;1}=\frac{r_{3}}{r_{1}}$$

(22) $$w_{3\;2}=\frac{r_{3}}{r_{2}}$$

Then we estimate the location as the point laying a distance *d*1 from the point *P*1 towards point *P*2 of the circle *C*1 and a distance *d*2 from there to the circle *C*2:

(23) $$d1=a\times w_{3\;1}\;$$

(24) $$d2=b\times w_{3\;2}\;$$

where *a* is the closest distance between the circumferences of *C*1 and *C*3, and *b* is the distance from the point resulting of moving from *P*1 to *P*2 and the closest point of the circumference *C*2.

The rest of the possible (but unlikely) cases are also implemented: circles intersect in pairs (i.e., *C*1 with *C*2 and *C*3 and no intersection between *C*2 and *C*3), two circles intersect and the third one is contained in any of the others but also intersecting the remaining one, two circles intersect and the third one is contained in any of the others but without intersecting the remaining one, the circles are contained each other (i.e., *C*1 inside *C*2 and *C*3, *C*2 inside *C*3), one circle isolated and one of the other ones inside the remaining one (i.e., *C*1 isolated, *C*2 inside *C*3).

### 3.5. Kalman Filtering

Once we have the estimated location from the weighted location algorithm, we intend to use the KF to smooth the calculations of the position. We have designed a second order filter to track position, velocity and acceleration in *x* and *y* axis, with no external control input. Through experimentation it was determined that the best values to initialize the filter are those detailed in Equations (25)–(30). We have to mention that tuning of the initial values is not a critical aspect since the KF adapts some of them over the time.

For the state variables *x*, we set them with the initial values of the variables we are measuring. In our case, for testing purposes, we set the position in *x* and *y* as the starting position of the device during the test. Velocity and acceleration are set to 0 as the initial values for our environment. Depending on the application, these values may change:

(25) $$x={\left[0\;0\;0\;0\;0\;0\;\right]}^{T}$$

Therefore, the used state transition function matrix ***F*** is:

(26) $$F=\left\{\begin{array}{cccccc} 1 & t & \frac{1}{2}t^{2} & 0 & 0 & 0\\ 0 & 1 & t & 0 & 0 & 0\\ 0 & 0 & 1 & 0 & 0 & 0\\ 0 & 0 & 0 & 1 & t & \frac{1}{2}t^{2}\\ 0 & 0 & 0 & 0 & 1 & t\\ 0 & 0 & 0 & 0 & 0 & 1 \end{array}\right\}$$

The state covariance ***P*** indicates how much the state variables influence the values each other. We have chosen medium values for the initial matrix ***P*** so that the system does not depend on the initial state values:

(27) $$P=\left\{\begin{array}{cccccc} 10 & 0 & 0 & 0 & 0 & 0\\ 0 & 10 & 0 & 0 & 0 & 0\\ 0 & 0 & 10 & 0 & 0 & 0\\ 0 & 0 & 0 & 10 & 0 & 0\\ 0 & 0 & 0 & 0 & 10 & 0\\ 0 & 0 & 0 & 0 & 0 & 10 \end{array}\right\}$$

Note: the columns are defined as position, velocity and acceleration in x and position, velocity and acceleration in y.

The process noise matrix ***Q*** is not updated in every step. Since we consider ***F*** to be accurate enough to define the movement process, the values of ***Q*** are defined small according to some performed empirical tests in all scenarios:

(28) $$Q=\left\{\begin{array}{cccccc} 0.1 & 0.1 & 0.1 & 0 & 0 & 0\\ 0.1 & 0.1 & 0.1 & 0 & 0 & 0\\ 0.1 & 0.1 & 0.1 & 0 & 0 & 0\\ 0 & 0 & 0 & 0.1 & 0.1 & 0.1\\ 0 & 0 & 0 & 0.1 & 0.1 & 0.1\\ 0 & 0 & 0 & 0.1 & 0.1 & 0.1 \end{array}\right\}$$

The measurement function H defines the mapping from the states variables to the measurements. *z* is the measurement vector, and x the states variables. The ***H*** function is used to obtain from the state variables vector *x* the values that are being measured, in this case the position:

(29) $$H=\left\{\begin{array}{cccccc} 1 & 0 & 0 & 0 & 0 & 0\\ 0 & 0 & 0 & 1 & 0 & 0 \end{array}\right\}\;z=Hx$$

Finally, the matrix ***R*** is related to the introduced noise in position measurement. We have performed some tests to tune this value, obtaining the best results for the matrix expressed in Equation (30):

(30) $$R=\left\{\begin{array}{cc} 4 & 0\\ 0 & 4 \end{array}\right\}$$

The Kalman gain *K* can be calculated as expressed in Equation (31):

(31) $$K=PH^{T}{\left(HPH^{T}+R\right)}^{-1}$$

All those parameters are combined during the prediction step, Equations (32) and (33), and the update step, Equations (33) and (34), to get the current state of the system:

(32) $${\hat{x}}_{k}=F_{k}{\hat{x}}_{k-1}$$

where ${\hat{x}}_{k}$ is the estimate of *x* at current step *k*:

(33) $$P_{k}=F_{k}P_{k-1}F_{k}^{T}+Q_{k}$$

where $P_{k}$ is the estimate of *P* at current step *k*:

(34) $${\hat{x}}_{k}^{\prime }={\hat{x}}_{k}+K\;\left({\vec{z}}_{k}-H_{k}{\hat{x}}_{k}\right)$$

(35) $$P_{k}^{\prime }=P_{k}-K\prime H_{k}P_{k}$$

where ${\hat{x}}_{k}^{\prime }$ and $P_{k}^{\prime }$ are the updated of *x* and *P* respectively at current step *k. K* is also updated in the *k* current step of the system, as showed in Equation (36), obtaining *K’*:

(36) $$K^{\prime }=P_{k}H_{k}^{T}{\left(H_{k}P_{k}H_{k}^{T}+R_{k}\right)}^{-1}$$

## 4. Test Scenarios

**Figure 6:**
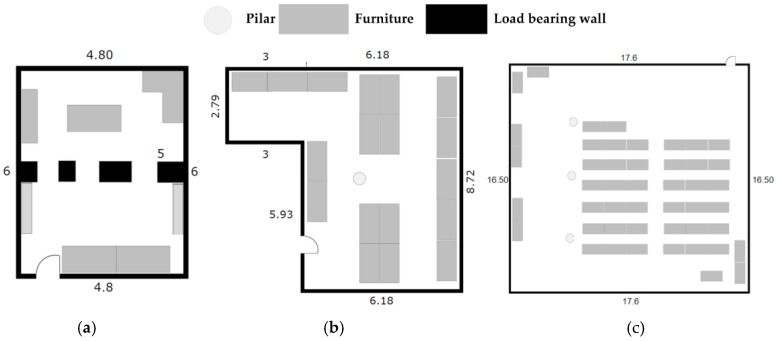
(**a**) Blueprint of scenario #1; (**b**) Blueprint of scenario #2; (**c**) Blueprint of scenario #3.

The objective of this implementation was to provide a system able to display good performance in terms of accuracy, cost and energy consumption in any environment. We have selected three different indoor scenarios, shown in Figure 6, to present the performance we have obtained.

- Scenario #1. Indoors medium sized room environment. There are not any obstacles between sender and receivers but we observe interferences from the WiFi and other electronic devices as well as refraction and multipath due to pillars and walls. Its size is a 6 m × 4.8 m.
- Scenario #2. Laboratory room. This is a laboratory full of computers, with people around using electronic devices with WiFi, Bluetooth, etc. causing interferences. Its size is 9.19 m × 6.18 m.
- Scenario #3. Conference room. Unlike scenario #2, now we have a bigger room where the receivers are further away than before. Its size is a 16.50 m × 17.60 m. The interferences are similar to those in Scenario #2.

All tests are performed in the presence of people, interference signals from electronic devices and rotating the BLE devices that act as senders since we know that the antenna is not fully omnidirectional. Therefore we perform tests in a realistic situation that can be considered as the worst case. The results we have presented are obtained in LOS conditions except for people presence. To extend our system in case we have no LOS, more receivers must be placed to obtain the same accuracy as is possible in a RSSI-based system. Tests are performed when senders and receivers are in the same plane. In the case this is not possible, then we have to apply 3D trilateration schemes not implemented in this version of the system, otherwise we must add a position estimation error. For a 3D location an additional reference is needed. This case has not been implemented since we consider this is not an interesting use case for our solution, since the attenuation due to floors introduces a significant error that can be sufficient to misplace the sender to a different floor level, becoming the location system useless. 

We have selected the parameters of the Log-Distance Path Loss model with shadowing and the ITU model according to the recommendations of [42] and [43], while for the empirical model we have selected A as the average RSSI value at the receiver one m away from the receiver after applying the biggest algorithm, and n is the experimental value that better matches the RSSI values. The equations for each model are described in Section 3.3, Equations (5), (6) and (7) respectively. The parameters are shown in Table 3, Table 4 and Table 5.

### 4.1. Estimation of the Most Accurate Propagation Model and RSSI Selection Algorithm

**Figure 7:**
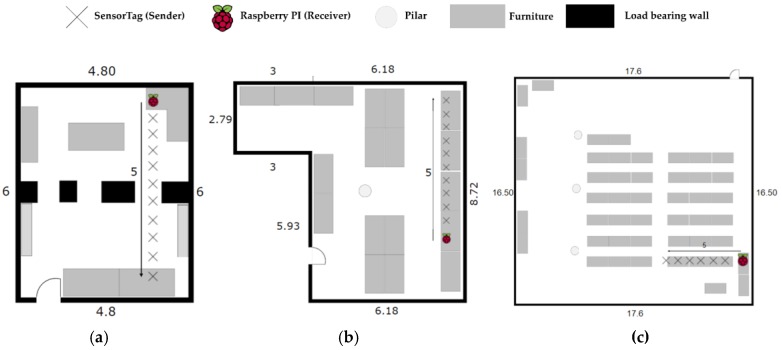
Scenarios to estimate the most accurate propagation model and combination scheme. (**a**) Scenario #1; (**b**) Scenario #2, (**c**) Scenario #3.

In order to evaluate the best propagation model and RSSI selection algorithm, we use scenarios #1 and #2. We have moved the sender away from the receiver from 0.5 to 5 m in steps of 0.5 m, taking 500 RSSI measurements at each step, as shown in Figure 7.

**Figure 8:**
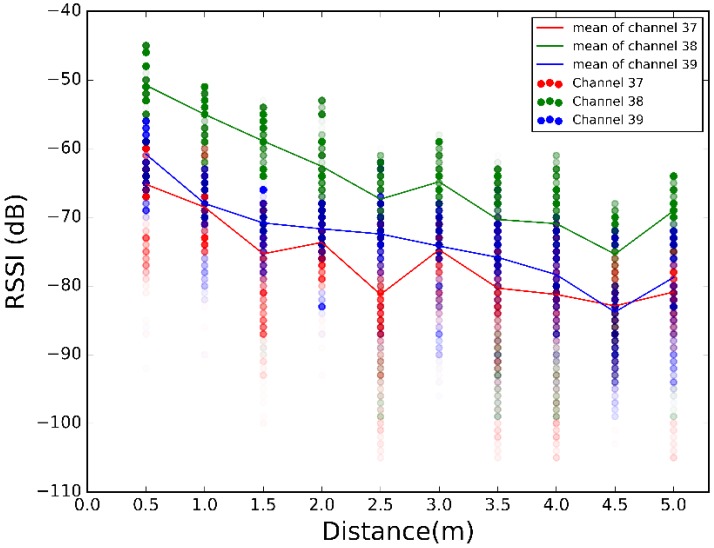
Scatter plot of RSSI measurements for different distances from sender to receiver at scenario #1 for different channels.

**Figure 9:**
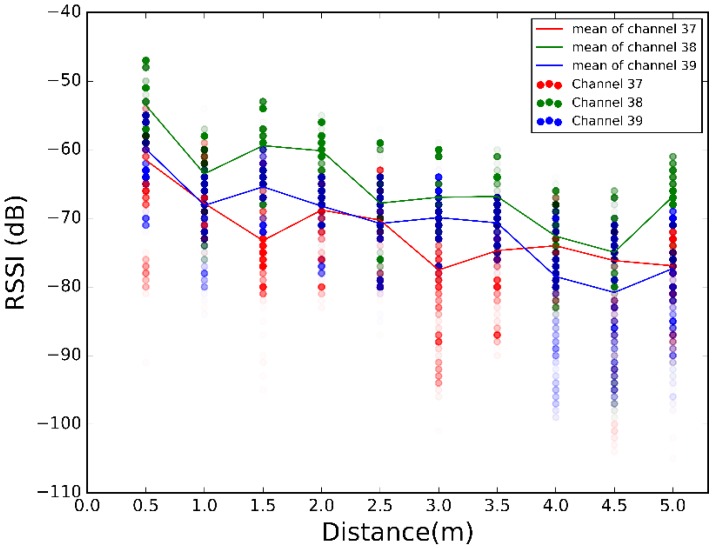
Scatter plot of RSSI measurements for different distances from sender to receiver at scenario #2 for different channels.

**Figure 10:**
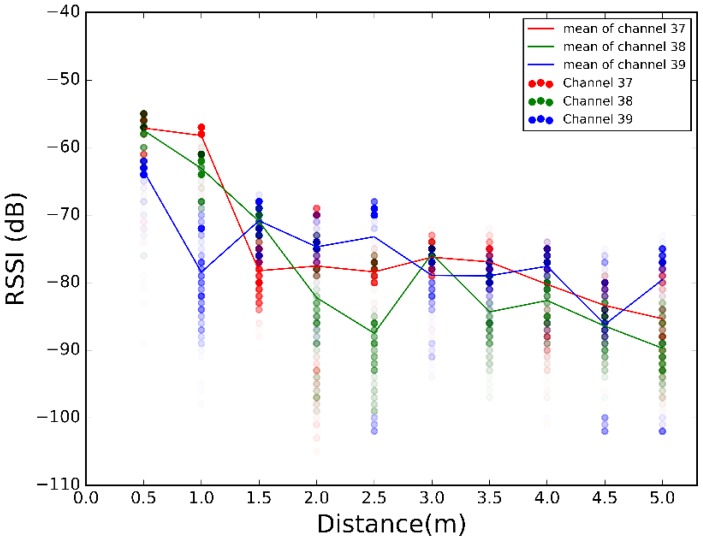
Scatter plot of RSSI measurements for different distances from sender to receiver at scenario #3 for different channels.

The scatter plots of the 500 RSSI values at each distance for each scenario are illustrated in Figure 8, Figure 9 and Figure 10. Plotting all the channels together, we have the overall dispersion and the mean for each channel.

**Figure 11:**
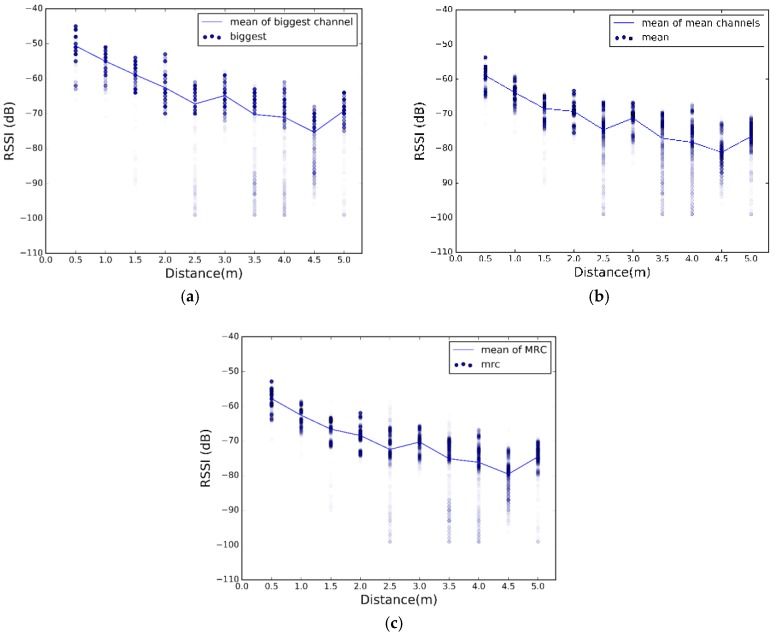
Scatter plot of RSSI measurements after applying combination schemes at scenario 1. (**a**) Biggest; (**b**) Mean; (**c**) MRC.

**Figure 12:**
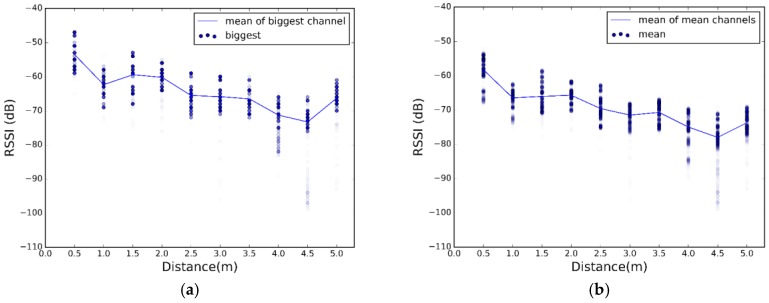

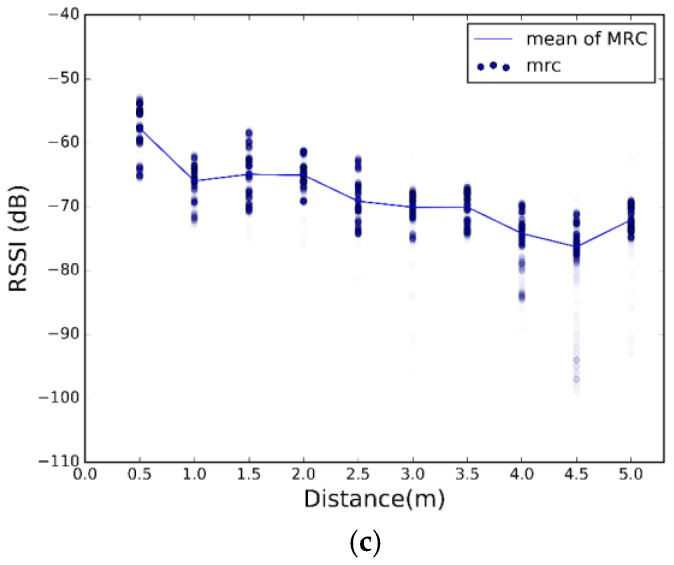
Scatter plot of RSSI measurements after applying combination schemes at scenario 2. (**a**) Biggest; (**b**) Mean; (**c**) MRC.

**Figure 13:**
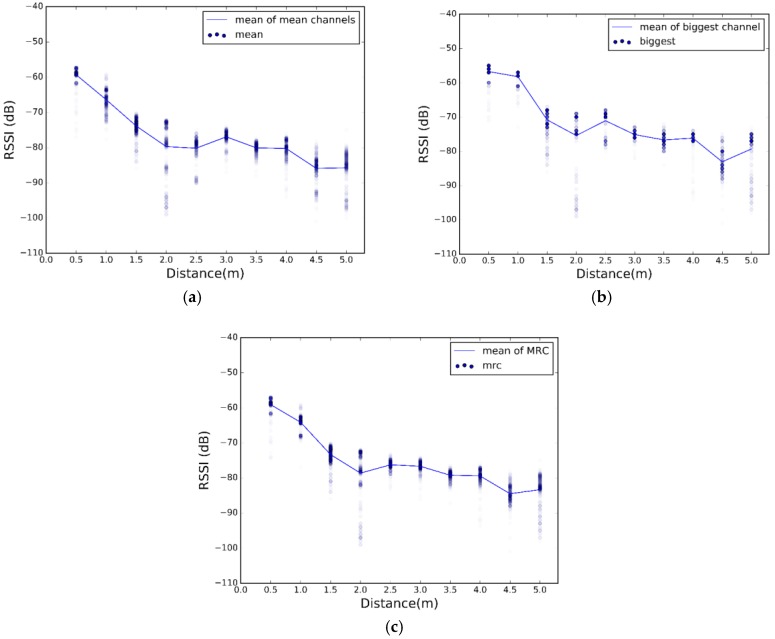
Scatter plot of RSSI measurements after applying combination schemes at scenario 3. (**a**) Biggest; (**b**) Mean; (**c**) MRC.

Figure 8, Figure 9 and Figure 10 show high fluctuations in RSSI, not only along the distances, but also within the same distance. These results prove the need of a combination scheme that reduces spread of the RSSI and ensures that the system uses the best channel to estimate the most accurate position. The results of applying the combination algorithms we are considering (detailed in Section 3.2.1), are shown in Figure 11, Figure 12 and Figure 13. In this case we have combined the three values of each row of Table 2 according to the biggest, mean or MRC algorithm in each case, to obtain the RSSI value presented in the Figure 11 and Figure 12 for each scenario.

Comparing Figure 11, Figure 12 and Figure 13 with Figure 8, Figure 9 and Figure 10, we visually observe that the difference between the maximum and minimum values of the RSSI measurement scatter plot is reduced when applying the different combination schemes over the three channels. Moreover, for example, for scenario #2, Table 6 shows the RSSI standard deviation as a dispersion measure. RSSI standard deviation is maintained or reduced in all the cases applying a combination algorithm. There are several techniques to use so as to minimize the fast fading effect, such as antenna diversity or frequency diversity. Thanks to the fact that the advertisement message is transmitted almost simultaneously in three different frequencies, we can apply the frequency diversity by free. The three combination schemes are commonly used against fast fading. As we compensate the fast fading, we reduce the variability of the RSSI. Therefore, we demonstrate that this combination has an impact on RSSI dispersion, and we conclude that we reduce the dispersion when applying the different combination schemes over the three channels. 

**Figure 14:**
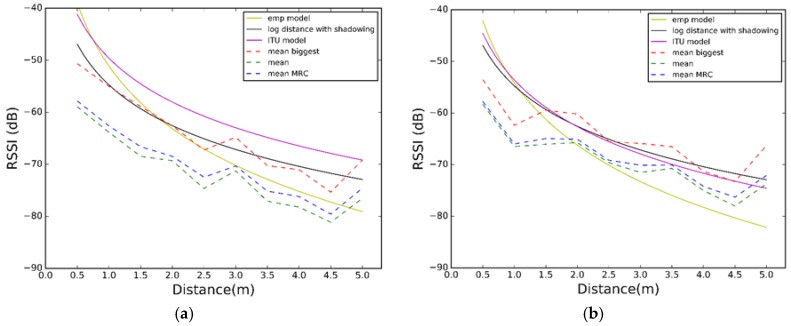

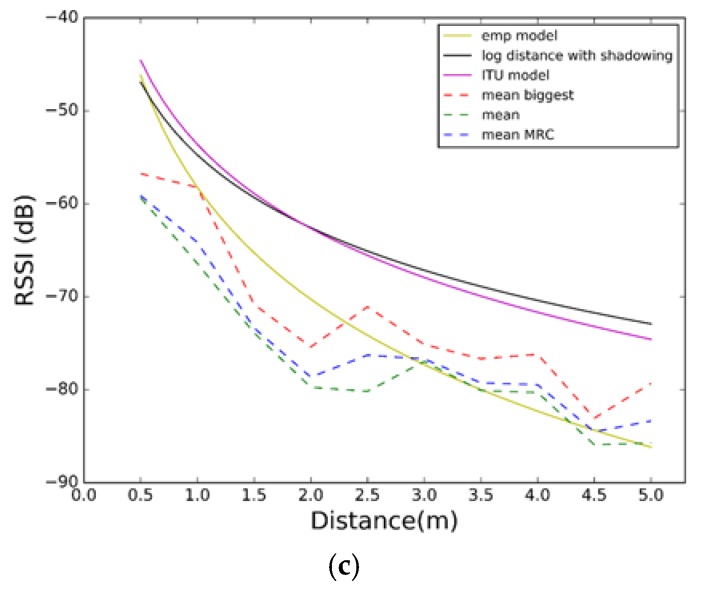
Comparison between the different propagation models and combination schemes. (**a**) Scenario #1; (**b**) Scenario #2; (**c**) Scenario #3.

Figure 14 shows the correlation between the propagation models and the combination schemes we have proposed. We observed that in scenario #1 the Log-Distance Path Loss model with shadowing fits better with the biggest algorithm than any other combination of propagation model and combination scheme. In scenario #2 there is almost no difference between ITU and Log-Distance Path Loss models with or without shadowing, and both perform well. Nevertheless, in scenario #3 we observe that the RSSI values are smaller and they do not match well with neither the ITU nor the Log-Distance Path Loss model. The best model the RSSI values match is with the empirical model, as expected since it considers some characterization. This last model is the one that performs the best.

**Figure 15:**
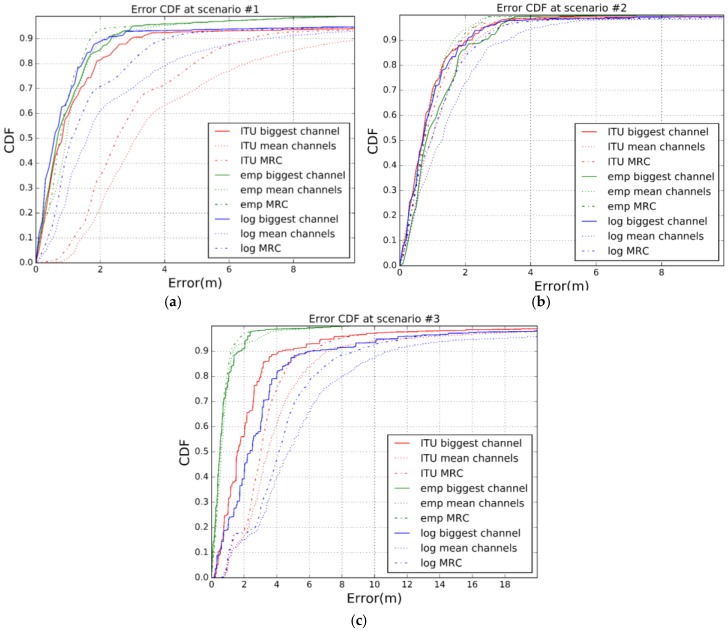
Error CDF for the different propagation models and combination schemes. (**a**) Scenario #1; (**b**) Scenario #2; (**c**) Scenario #3.

However, to be precise, we want to check the position estimation error for any combination of propagation model and combination scheme. According to Equation (1), we have computed the Cumulative Distribution Function (CDF) of the error average over all the distances from 0.5 m to 5 m. Figure 15 shows the results. CDF represents the probability that the error average takes a value less than or equal to the error average in meters. From Figure 15 we observe that, as expected, the best propagation model is the empirical one. However, the improvement in the accuracy is not as high as the drawback, in terms of characterization, that this propagation model entails. Respect to the other two models, ITU and Log-Distance Path Loss model with shadowing, we came around a dilemma. In scenario #1 the Log-Distance Path Loss model achieves a better accuracy than the ITU model (2 m during 90% of the time against 3 m during 90% of the time). In scenario 2 there is almost no difference between models, but in scenario #3, the ITU model is the one outperforming the Log-Distance Path Loss model for around 2 m (ITU gets 4 m accuracy 90% of the time while Log-Distance Path Loss model with shadowing gets an accuracy of 6 m during 90% of the time).

Since one of our main objectives is to build a system as independent of the environment as possible, we recommend using the Log-Distance Path Loss model with shadowing. While the ITU model considers different values for its parameters N and $P_{f}\left(n\right)$ according to the type of the building, the number of floors it has, and the frequency, the Log-Distance Path Loss model with shadowing takes its parameters n and Xσ depending on the frequency and the type of the building. 

Therefore, from now on, the *Log-Distance Path Loss* model with shadowing and the *biggest RSSI algorithm* are the propagation model and the channel diversity combination scheme we are going to use, respectively.

### 4.2. Performance of the System

Once we have selected the propagation model and the combination scheme, we must measure the performance, by means of position error, of the overall system. In order to do this we have selected scenarios #2 and #3, as they have the same interference conditions, to check the behaviour of the system in two different sized indoor environments. In this case, we have placed four receivers in each scenario. In the trilateration algorithm, we have used the three receivers whose calculated distance to the sender is the smallest, according to our approach of trusting more the devices closer to the sender. Using more than three receivers increases the accuracy, but also increases the cost of the system.

The performed tests will calculate the position of the senders every time interval. This time interval can be greater than the advertisement interval (in the tests set to 100 ms). The results presented calculate the position of each sender every second. This is why we have up to N = 10 rows of the matrix of Table 2 to estimate the sender position. Therefore, we can decide how to use this amount of data. For testing purposes we use the measurements of seven rows (21 RSSI measurements), to obtain an optimized RSSI each second. This value has been chosen as a compromise of accuracy, used memory and delay in the position computation.

**Figure 16:**
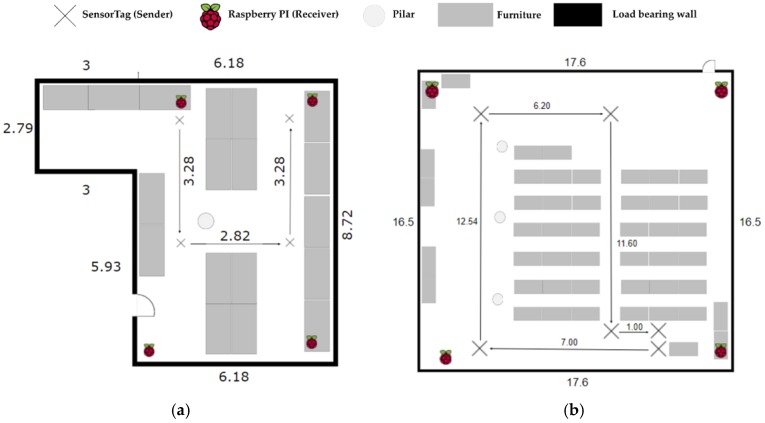
Path followed for the different scenarios. (**a**) Path at scenario #2; (**b**) Path at scenario #3.

The procedure is as follows: we start from a given position and we move with the sender at a constant velocity of 0.3 m/s following a defined path in each scenario as shown in Figure 16, with the purpose of calculating the CDF. Then we compare the estimated positions with the one we should be at each second and perform the CDF of the averaged error.

To show the improvements of the different proposals, we have tested the system using raw values (i.e., without diversity, without KF, without weighted trilateration or a combination of those techniques), only diversity, diversity plus weighted trilateration or KF, and finally all of them together. We have performed four trials for each technique to compute the averaged results. In case we do not use diversity, we have selected one channel randomly, and to compute the position we have used the basic trilateration algorithm. Table 7 shows the results for the 90% and 95% in both scenarios.

**Figure 17:**
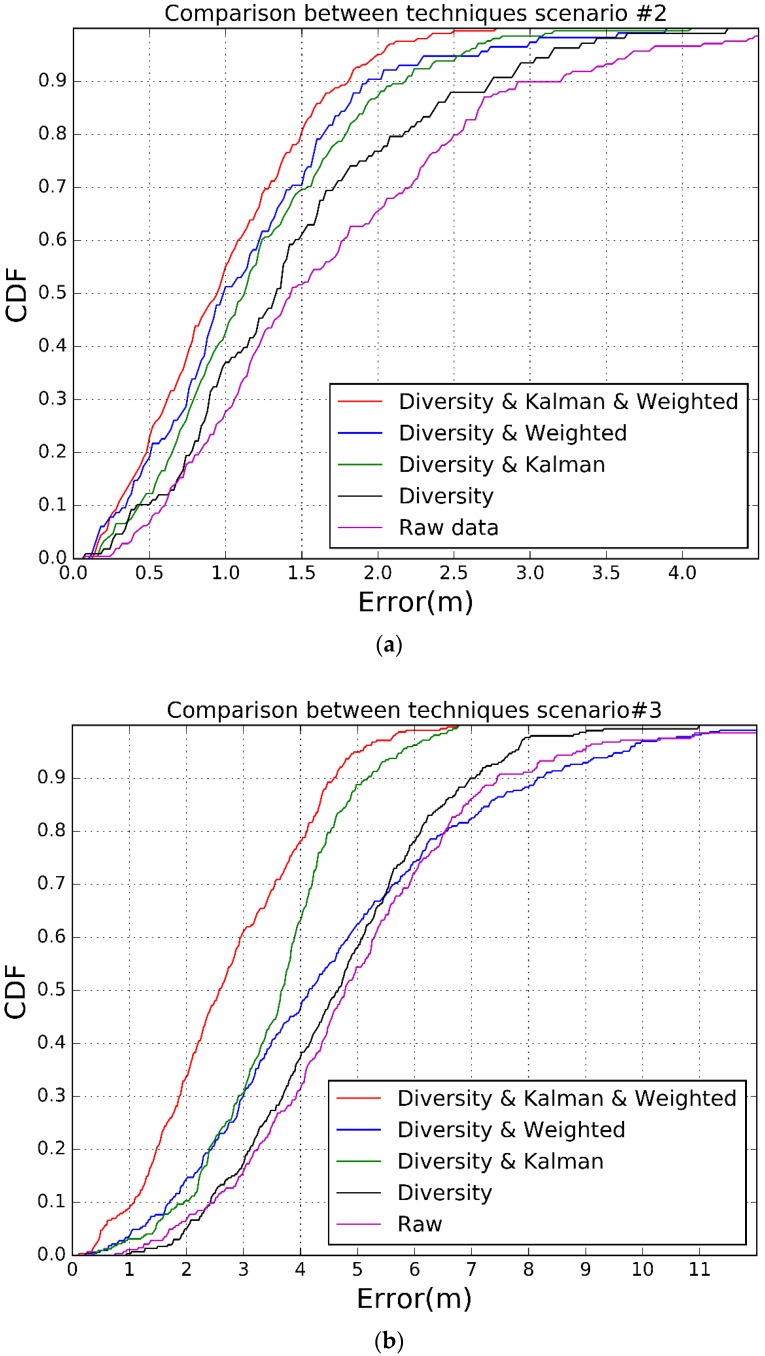
CDF. (**a**) Scenario #2; (**b**) Scenario #3.

Figure 17 shows that our proposal, that includes channel diversity, Kalman filtering and weighted trilateration, improves the estimation error compared to the other approaches. In scenario #1, an improvement of 43.47% is achieved when comparing errors at 90% of the time. Furthermore we observe that, while the error in scenario #2 is low, below 1.82 m during 90% of the time, in scenario #3 the error goes up to 4.6 m during 90% of the time. This issue is due to the fact that accuracy of the RSSI measurements decreases drastically as the distance between the sender and the receiver increases from 5 m on. This is a design trade-off so that we could decrease the error by adding more receivers if the application requires greater accuracy, but increasing the cost.

### 4.3. Power Consumption Analysis and Application Examples

Apart from performance and location error, the device lifetime is one of the most important aspects in a system like the one we propose. For this reason, in this section we do an estimation of the power consumption of the system. Texas Instrument has developed a tool [44] to do an estimation of the power consumption of SensorTag CC26xx series. For a device with a power source voltage of 3 V, a CR2032 coin cell battery, considering only non-connectivity advertising at 100 ms 24 h/day, with an output power level of 5 dBm (the maximum) and advertising in the three BLE channels using frames of 30 bytes, we show the current consumption and device lifetime estimation in Table 8.

Depending on the user application, the advertising interval can be greater or smaller. However, 100 ms advertising interval is already a high value. Table 9 shows current consumption and device lifetime for applications where the advertising requirements are not so high and the BLE tag can advertise each 500 ms.

Different applications and environments under several conditions where our system may be useful as well as the device lifetime, are explained as follows:

- Tracking assets in a factory where workers move them from one building to another, and they want to track that all the assets are where they are supposed to be. Each asset must have a BLE tag on it or in its container. If we want to track in which building the assets are at every moment, we need to have the device on 24 h but we do not need an extreme location precision, so we transmit advertisements from the BLE tag every 5 s instead of every 100 ms. Under these conditions (24 h running, 5 s advertisement, low transmission power of −20 dBm), the assets could stay monitored for 6.238 years.
- Tracking people inside a building where, for security reasons, we want to have the control of where the people are at every moment. People must have a BLE Tag. We have considered a real operation time of the system of 10 h per day. In addition, we know we will have many interferences and occlusions from people moving and the devices they carry, so we need to set the advertising interval to a low value (100 ms) and the transmission power to 5 dBm so that the accuracy does not drop. In this case the device lifetime is about 4.2 months.
- Tracking customers in a mall to know users’ preferences and offer them the products that they are interested in. The IPS may be used to track the path of the customers. With the information obtained, companies of the mall may, for example, redistribute the different shops in a way that is more comfortable for the customers, or place together shops that are usually visited in a row. Taking into account that malls usually open 12 h per day, and that we need a medium precision for this purpose (500 ms advertisement interval and medium transmission power of 0 dBm), we obtain a device lifetime for the BLE tags of 1.4 years.

Moreover, the BLE SensorTag chosen for this project can be programmed so that it stops advertising frames when it is not in movement.

## 5. Conclusions

In this paper we have presented a survey of different IPS BLE based systems. Then we have proposed and implemented a novel IPS BLE system that improves accuracy while reducing costs. In our research, we have proposed three different techniques to enhance the precision of a BLE indoor positioning system: channel diversity, Kalman Filtering and a weighted trilateration method. With channel diversity our main goal is to reduce the dispersion of the RSSI measurements inherent to this kind of systems. We use Kalman Filtering as a way to mitigate the effects of unlikely or impossible location estimations due to wrong RSSI measurements, so that we can track the location of a device with more precision. Finally, the weighted trilateration is an improvement of the basic trilateration algorithm, since not always the three measurements converge to a single point. Our results prove that combined together, the precision is increased by 43.47% in a medium-size room scenario and by 38.33% in a big-size room scenario compared to precision without using any of the proposed techniques.

If we compare the results of our experiments to those reviewed in the literature, we observe that most of the systems use fingerprinting, since it is the technique that achieves the best results in terms of precision. For example, [19] shows a precision under 0.8 m during 96.6% of the time which is far better than the results of our proposal at 95%, of two m in scenario #2 and 5.06 on scenario #3. Nevertheless, there are two important factors which make our system a good choice: the number of beacons and the characterization of the scenario. In [19], they use 44 beacons which implies a more expensive solution compared to our four beacons. In addition to the cost, their system is highly dependent of characterization, as any other fingerprinting approach, while our proposal does not need any characterization. 

When comparing our solution to other BLE systems using propagation models without characterization, we observe that, in general, ours is the most balanced one in terms of precision and cost. Comparing with [11] we see that our proposal outperforms it in every metric, namely number of beacons, size of the room, and accuracy. Finally, the authors in [17] used 10 beacons in a bigger room than ours in scenario #3, and the precision was 3.02 m during 80% of the time. In our case, the precision in scenario #3 is around 4 m during 80% of the time. However, we still have to consider that they are using six more beacons that, again, increase the final cost. As the result of applying all the techniques proposed in our work, we achieve an estimation error for a device moving lower than 1.82 m during 90% of the time for a 54 m^2^ room, and lower that 4.6 m during 90% of the time for a 290 m^2^ room, in both cases using only 4 beacons. An estimation of the cost of BLE devices used in the system is between 15€ to 30€ each BLE beacon (one per sender), and about 120€ per each receiver. The comparison of the system we have proposed with other studies has proved that our solution is better as a trade-off between precision and cost, as the density of receivers is very low for the accuracy achieved. From the figures provided it is clear that if we have a large number of low power tracking devices, which should work unattended, the proposed solution is the best in terms of cost.

## Figures and Tables

**Table 1:** Sum up of most relevant related work. “-“ when information is missed.

Reference	Scenario (m × m)	Number of Beacons	Precision (%Time and Meters)	Technology/Used Methodology/Algorithm (When Specified)
[6]	12 × 3	-	99% below 1.68 m	-/Inertial navigation/-
[19]	10.5 × 15.6	44	96.6% below 0.8 m	BLE/Fingerprinting/-
[11]	6 × 6	8	75% below 1.8 m	BLE/-/Stigmergy and Min-Max/
[20]	44 × 22	9	1.58 m total averaged	BLE/Fingerprinting/-
[18]	45 × 12	19	95% below 8.5 m (WiFi) 95% below 2.6 m (BLE)	BLE/WiFi/Fingerprinting/-
[13]	unknown	13	Unknown/Only whether a device is in a room or not	BLE/-/KF
[21]	16.8 × 12.6	10	90% below 4.12 m	BLE/Fingerprinting/-
[31]	17.5 × 9.6	10	90% below ~2 m	BLE/Fingerprinting/Deep learning
[28]	10 × 7	3	Unknown/Probability of true localization	BLE/Fingerprinting/Ray launching based simulation model
[22]	160 m^2^	4	2.33 m	BLE/Fingerprinting and WiFi/-
[37]	1.5 × 12	3	90% below 3 m	BLE/-/Particle filtering
	8 × 6	4	90% below 4 m	BLE/Fingerprinting/-
[32]	3.6 × 20	6	90% below 2.25 m	BLE/Fingerprinting/RSS Feedbacks
[24]	9.3 × 6.3	5	Unknown/Probability of being in a given sector	BLE/Fingerprinting/Transmission power settings
[23]	40 × 8	7	90% below 3.58 m	BLE and WiFi/Fingerprinting/-
[17]	32.5 × 19.2	10	80% below 3.02 m	BLE/-/Adaptive multi-lateration
[27]	100 × 100	From 10 to 100	From 5 m to 50 m averaged	BLE/Fingerprinting/-
[15]	12 × 3	3	Unknown/Only whether a device is in a room or not	BLE/-/-
[10]	1200 m^2^	6	90% below 3.8 m	BLE/Machine learning/-
[16]	3.6 × 15	8	1 m averaged	BLE/-/KF, dead reckoning
[29]	60 × 40	20	90% below 2.57 m	BLE/Fingerprinting /Polynomial Regression model, Extended KF, Outlier Detection
		8	90% below 4.16 m	

**Table 2:** Circular matrix for RSSI values, for each sender.

Counter ID	Channel 37	Channel 38	Channel 39
1	$RSSI_{37}$	$RSSI_{38}$	$RSSI_{39}$
2	$RSSI_{37}$	$RSSI_{38}$	$RSSI_{39}$
…	…	…	…
N	$RSSI_{37}$	$RSSI_{38}$	$RSSI_{39}$

**Table 3:** Log-Distance Path Loss model with shadowing.

Scenario	N	Xσ
1	2.6	14.1
2	2.6	14.1
3	2.6	14.1

**Table 4:** ITU model for indoor environment.

Scenario	N	P_f_(n)	n
1	28	10	1
2	30	14	1
3	30	14	1

**Table 5:** Empirical model.

Scenario	n	A
1	4	−51.12
2	4	−54.18
3	4	−58.22

**Table 6:** RSSI standard deviation for scenario #2.

Distance in Meters										
	0.5	1	1.5	2	2.5	3	3.5	4	4.5	5
**CH 37**	6.59	4.58	5.45	4.52	4.72	7.09	5.61	4.25	6.48	4.54
**CH 38**	3.79	4.41	4.83	2.85	5.42	4.26	3.41	4.68	6.35	4.01
**CH 39**	4.79	4.38	3.37	3.51	4.78	3.42	2.76	6.95	8.01	6.84
**Biggest**	3.79	3.15	4.89	2.93	3.57	3.9	2.92	4.41	5.83	3.08
**Mean**	4.01	2.51	3.81	2.39	3.59	2.74	2.87	3.92	5.26	2.88
**MRC**	3.67	2.48	3.28	2.19	3.37	2.4	2.44	3.66	5.06	2.34

**Table 7:** Precision comparison.

Technique	Error(m)			
	Scenario 2		Scenario 3	
	90% of Time	95% of Time	90% of Time	95% of Time
Raw	3.22	3.8	7.46	8.84
Diversity	2.76	3.14	7.08	7.78
Diversity & Kalman	2.18	2.56	5.18	5.78
Diversity & Weighted	1.94	2.68	8.16	9.66
Diversity & Kalman & Weighted	1.82	2.0	4.6	5.06

**Table 8:** Current consumption and device lifetime when considering only non-connectivity advertising at 100 ms 24 h/day, with an output power level of 5 dBm.

Days	Months	Years	Peak TX Current [mA]	Average Current [mA]
53.138	1.771	0.146	9.3	0.18035

**Table 9:** Current consumption and device lifetime when considering only non-connectivity advertising at 500 ms 24 h/day, with an output power level of 5 dBm.

Days	Months	Years	Peak TX Current [mA]	Average Current [mA]
259.923	8.664	0.712	9.3	0.03687
